# Do Mobile Phone Surveys Supplement Other Mortality Surveillance Methods? Results From a National Study in Bangladesh

**DOI:** 10.1111/tmi.70181

**Published:** 2026-06-19

**Authors:** Aashish Gupta, Gulam Muhammed Al Kibria, Tahsin Shahrin Khan, Chandra Nirod Saha, Farzana Islam Khan, Alain Labrique, Tahmina Shirin, Mohan Diwakar, Georges Reniers, Iqbal Ansary Khan, Dustin G. Gibson, Stephane Helleringer

**Affiliations:** ^1^ Division of Social Science New York University – Abu Dhabi Abu Dhabi UAE; ^2^ National Institute on Aging Bethesda Maryland USA; ^3^ Institute of Epidemiology, Disease Control, and Research Dhaka Bangladesh; ^4^ World Health Organization, Department of Digital Health and Innovation Geneva Switzerland; ^5^ Johns Hopkins University, Bloomberg School of Public Health Baltimore Maryland USA; ^6^ London School of Hygiene and Tropical Medicine Department of Population Health London UK

**Keywords:** Bangladesh, COVID‐19, epidemics, mobile phones, mortality, surveillance, surveys

## Abstract

**Objectives:**

To test whether mobile phone surveys conducted remotely might generate data on deaths that are not covered by mortality surveillance systems established in laboratories and health facilities, or in institutions involved in the post‐mortem management of corpses (e.g., morgues, cemeteries).

**Methods:**

We conducted a national survey of mobile phone users in Bangladesh during the COVID‐19 pandemic. Respondents were asked to list the deaths that had occurred in their household in recent years. For each death, they were also asked to indicate whether the deceased had been diagnosed with COVID‐19 prior to death, whether the death occurred at home or at a health facility, and where the burial took place. Using these data, we represented the overlap between data generated by the mobile phone survey and several potential mortality surveillance systems, which focus on counting deaths in health facilities or communal cemeteries. We described the socio‐demographic characteristics of the deaths that were uniquely recorded by the mobile phone survey.

**Results:**

From December 2021 to July 2022, 506,659 calls were placed, resulting in 22,731 interviews completed by mobile phone. Respondents reported 1527 deaths that had occurred in their household since 2020. Among those deaths, 99 (6.5%) had received a pre‐mortem diagnosis of COVID‐19. The proportion of reported deaths that were not covered by other potential mortality surveillance systems, was 32.4% in the large city corporations of Dhaka and Chittagong, 36.3% in other urban areas and 49.6% in rural areas. In urban areas, deaths among men, as well as among youth and the elderly, were more likely to be covered solely by the mobile phone survey.

**Conclusion:**

Mobile phone surveys can potentially remedy gaps in the data generated by other recommended mortality surveillance methods.

## 1 Introduction

In many low‐and‐middle income countries (LMICs), civil registration and vital statistics (CRVS) systems are too incomplete to monitor mortality fluctuations in real‐time [1]. Investments to strengthen CRVS are under way [2, 3], but they will require sustained investment to yield sufficient levels of registration. In the meantime, alternative methods of mortality surveillance are needed to increase the amount and timeliness of available data on deaths, and to ensure that crises, such as those prompted by epidemics, conflicts or climate events, are rapidly detected and addressed in LMICs [4, 5].

Household surveys are the main interim source of data on mortality in LMICs. They ask respondents to report recent deaths among members of their household, or among some of their relatives [6]. Household surveys are used by governments and international organizations to estimate mortality rates at the national level [7, 8] but they are not well‐suited to monitor short‐term mortality fluctuations. In most LMICs, household surveys take place every 3–5 years. This periodicity implies that the impact of a disease outbreak, disaster or conflict might not be documented for several months or even years after it has begun.

In many LMICs, surveillance systems might also generate data on all‐cause mortality periodically [9]. Such systems might focus on health facilities, where health information systems count deaths among inpatients. They might also be implemented in settings where the post‐mortem management of corpses takes place, including morgues, cemeteries or coffin workshops [10, 11, 12, 13]. Conducting mortality surveillance in such places requires data entry of existing registers, and/or posting dedicated staff to record events [14, 15]. It might also rely on newer technologies. For example, satellite images allow monitoring burials at specific cemeteries [16, 17]. Other investigations have observed obituaries posted on public websites or tracked the volume of internet searches related to funerals [18, 19].

All‐cause mortality surveillance (ACMS) systems have recently documented periods of excess mortality—that is, an increase in deaths beyond the number of deaths that would have been expected based on historical trends [20]—in several localities during the COVID‐19 pandemic [21, 22]. Most existing ACMS implemented in LMICs are however partial. They are concentrated in urban centres, and seldom cover rural areas, or the rapidly expanding peri‐urban zones that border metropolises. They also include only a selective subset of the deaths that have occurred in a population, such as those that took place at health facilities, or that were buried at municipal cemeteries. Yet, in LMICs, large segments of the population might die at home [23, 24], and might be buried on family‐owned plots of land. Finally, the data produced by ACMS systems might be confounded if health‐seeking behaviours and/or post‐mortem practices change over time. For example, whereas significant numbers of urban residents in LMICs might traditionally be buried in their rural village of origin, this might not be feasible during epidemics or conflicts, due to restrictions on personal travel and the transportation of corpses. Bereaved families might also hesitate to post an obituary on a public website during an epidemic, if the circulating disease carries stigma. In such instances, fluctuations in the number of deaths recorded by ACMS systems might not only reflect excess mortality, but also changes in the coverage of surveillance systems.

Other ACMS systems address some of these limitations. Health and demographic surveillance systems (HDSS) and sample registration systems (SRS) actively track recent deaths, irrespective of their cause or place of occurrence [25, 26, 27, 28, 29, 30, 31]. To do so, they employ field workers who conduct regular household visits. They also mobilize community informants, who maintain lists of recent deaths to be investigated. Whereas HDSS are often focused on small populations, SRS monitors mortality in a random sample of clusters that are disseminated throughout broader areas and are representative of large populations of interest. However, SRS have only been implemented in a limited number of countries, due primarily to high set‐up costs and requirements for sustained funding [32, 33].

During the course of epidemics, case‐based surveillance systems might at times be activated to count deaths linked to the circulating disease. Such systems were put in place in west African countries during the 2013–2016 outbreak of Ebola virus disease [34], and in many LMICs during the COVID‐19 pandemic [35, 36]. Unfortunately, case‐based surveillance systems require extensive capacity to diagnose an illness. In LMICs where laboratory infrastructure is limited, these systems might miss deaths that occur among people who suffer from a disease but have not been adequately tested [37]. They might also fail to count deaths among people who have been diagnosed with a disease but later died at home, without follow‐up [38].

In this paper, we assess whether mobile phone surveys (MPS) can potentially address some of the gaps that affect other mortality surveillance systems, such as the ACMS systems established at health facilities or in institutions involved in the post‐mortem management of corpses, or the case‐based surveillance systems that focus on specific diseases. In MPS, respondents are contacted and answer questions entirely remotely, by mobile phone [39]. The costs of conducting MPS are much lower than those associated with in‐person data collection [40]. MPS can thus be repeated more frequently than household surveys. Due to the increased penetration of mobile phones throughout LMICs, MPS might also adequately reach large segments of various populations of interest, especially if they implement sampling quotas [41].

Mortality‐related MPS have been conducted during epidemics and conflicts [42, 43, 44] when household surveys were interrupted because in‐person interviews put interviewers and respondents at risk of disease and/or injury. Evaluations of MPS data on mortality have focused on internal consistency checks [45] and aggregate comparisons with reference estimates of mortality levels, such as those compiled by agencies of the United Nations [46, 47]. They have not investigated whether mortality‐related MPS document deaths that are not already covered by other surveillance systems. We analysed data from a unique national mortality‐related MPS from Bangladesh, which collected data on places of deaths and post‐mortem practices. It also inquired about pre‐mortem COVID‐19 diagnosis. These data allowed determining whether deaths reported during an MPS might also have been covered by various potential case‐based and ACMS systems.

## 2 Data and Methods

### 2.1 Study Setting

Bangladesh is a lower‐middle‐income country of more than 170 million inhabitants, located in south Asia. Prior to the COVID‐19 pandemic, it had an estimated life expectancy of approximately 73 years. A case‐based surveillance system established early during the pandemic recorded 29,500 COVID‐related deaths, that is, approximately 17.3 deaths per 100,000 person‐years [48]. The impact of the COVID‐19 pandemic has been measured through household surveys conducted in parts of the country [49]. Several ACMS systems also documented periods of excess mortality during the first 2–3 years of the pandemic. For example, the surveillance of burial sites in Dhaka detected significantly more deaths in 2020 than in 2018/19 [50, 51]. In a rural HDSS, excess mortality was concentrated among the oldest age groups of the population in the first months of the pandemic [52] whereas the national SRS indicates that life expectancy declined in Bangladesh by a few months in 2020, before rebounding for men, but not women [53].

Access to mobile phones is common among the population of Bangladesh. According to the most recent Demographic and Health Survey, almost all households have at least one mobile phone. Personal ownership of mobile phones varies by gender and residence. In urban areas, ⅔ of women and 85% of men aged 13 years and older owned a mobile phone in 2022. In rural areas, corresponding figures were 51% and 78%, respectively [54]. Several MPS focused on health issues have been conducted prior to the COVID‐19 pandemic in Bangladesh [55, 56]. Since then, a sample of households in 135 rural villages was enrolled in a MPS to measure changes in mortality and household income precipitated by the pandemic [57]. Hotlines and mobile applications were also used to monitor the occurrence of COVID‐related symptoms throughout the country [58].

### 2.2 Data Source

As part of the Rapid Mortality Mobile Phone Surveys (RaMMPS), the Institute of Epidemiology, Disease Control and Research (IEDCR) conducted a nationwide MPS between December 22, 2021 and July 31, 2022. Respondents were mobile phone users who had been recruited by random digit dialling [59]. Specifically, a series of potential mobile phone numbers was randomly generated using the prefixes and numbering schemes for all four mobile phone operators in Bangladesh. Then, trained data collectors from IEDCR's call centre dialled the randomly selected phone numbers and recorded call outcomes. Upon answering, mobile phone users were given a brief introduction to the study and asked if they wished to continue; if they declined, the call was ended.

Those interested in the study were asked screening questions. Only mobile users who were aged 18 years and older were eligible to participate. The recruitment process involved active monitoring across 24 sampling strata, defined by participants' age, gender, and place of residence [41]. Age of the mobile user was classified into 4 groups: 18–29 years old, 30–39 years old, 40–49 years old and 50 years and older. Residence was classified into three groups: residents of the large city corporations of Dhaka and Chittagong (DCC, CCC); other urban residents and rural residents. The enrolment target was 1040 0r 1045 respondents in each stratum, for a total of 25,000 planned participants. Mobile users who were reached after the target enrolment for their stratum had been achieved were deemed ineligible.

After confirming eligibility, interviewers sought the verbal consent of mobile users to participate in the MPS. To document consent, interviewers were required to check a series of boxes indicating that (1) detailed information about the survey was read to the respondent; (2) the respondent was provided the opportunity to ask any questions they had, and (3) the respondent had given his/her verbal agreement to participate in the interview. Interview questions covered characteristics of the respondents themselves (e.g., educational level, occupation), as well as information about the characteristics of their households (e.g., number of members). Respondents were also asked if there had been any deaths among members of their household since 2019, and if so, how many such deaths had occurred. For each death, data collectors asked respondents to state the gender, age at death and marital status of the deceased, as well as the month and year of occurrence.

Our study focuses on the deaths that happened since 2020. For this subset, data collectors asked questions about circumstances of the death, including (1) where it had occurred (at a health facility vs. at home), (2) where the deceased had been buried (in a communal cemetery, on a family plot or elsewhere) and (3) whether the deceased had received a COVID‐19 diagnosis prior to death. These variables indicate whether a death might have been recorded by one or more potential mortality surveillance systems. For example, deaths in which the deceased was diagnosed with COVID‐19 pre‐mortem might be recorded by a case‐based surveillance system, whereas deaths that were buried in a communal cemetery might be recorded by a potential ACMS system that covers this burial ground. Data collection procedures were approved by institutional review boards at Johns Hopkins University (Baltimore, USA) and the Institute of Epidemiology, Disease Control and Research (Dhaka, Bangladesh).

### 2.3 Analysis

We first described the mobile users who participated in the MPS. We compared the socio‐demographic characteristics (e.g., age, gender, marital status) of respondents who reported that a death had occurred in their household since 2020 to those of other respondents. We used 𝜒^2^ tests to assess whether the distribution of these socio‐demographic characteristics varied between respondents with and without a death in their household since 2020. Second, we investigated the characteristics of the deaths reported by MPS respondents, such as the gender, age at death, and marital status of the deceased. We also considered variables denoting the places of death and burial of the deceased household members, as well as an indication of a pre‐mortem positive COVID‐19 test.

Third, we plotted the joint distribution of place of death, place of burial and prior COVID‐19 diagnosis among the deaths reported during the MPS. We used proportional Venn diagrams to represent the extent of overlap between the different categories of these variables. Each Venn diagram is framed by a rectangle that represents all deaths reported during the MPS in a specific (sub‐)population. It also includes 3 possibly intersecting circles, whose total surface is proportional to the number of deaths that occurred at a health facility, were buried at a communal cemetery, and received a pre‐mortem COVID‐19 diagnosis, respectively. The area of the rectangle that is not occupied by one or more of the circles represents deaths that could not be detected by case‐based surveillance of COVID‐19 deaths or potential ACMS systems, and thus could only be detected by an MPS. We used the PVENN module in Stata to construct these visualizations [60].

Finally, we reported the characteristics of deaths that could only be detected by an MPS, and not by potential case‐based and ACMS systems. We explored correlates of the likelihood that a death was solely listed during the MPS. To do so, we used logistic regression models, in which the dependent variable was a binary variable taking value 1 if the death was only listed in the MPS, and 0 if it might also have been recorded by other potential mortality surveillance systems (i.e., case‐based or ACMS). Independent variables in these regression models included characteristics of the deceased such as his/her gender, age at death and marital status.

To better understand whether MPS might supplement other mortality surveillance systems in under‐served areas of the country, we conducted all our analyses separately by place of residence, that is, one of the three characteristics used to stratify the sample of MPS respondents. We assessed through 𝜒^2^ tests whether the distribution of socio‐demographic characteristics of the deceased, and circumstances of their deaths, varied between large city corporations (e.g., Dhaka), other urban areas and rural areas. Within each of these residential strata, we only conducted unweighted analyses of study data because the likelihood of reporting a death was not associated with the other two stratifying variables used for sampling MPS respondents (i.e., their gender and age, see Table 1 below).

**TABLE 1:** Characteristics of MPS respondents, by residence and reporting of recent household deaths.

	Large city corporations, Dhaka and Chittagong (%)			Other urban areas (%)			Rural areas (%)		
	No deaths since 2020	At least 1 death since 2020	*p* ^c^	No deaths since 2020	At least 1 death since 2020	*p* ^c^	No deaths since 2020	At least 1 death since 2020	*p* ^c^
*N* ^a^	5436 (94.4%)	321 (5.6%)	—	7905 (92.8%)	617 (7.2%)	—	7863 (93.0%)	589 (7.0%)	—
Gender^a^			0.522			0.067			0.316
Male	3202 (94.7%)	179 (5.3%)		4260 (93.4%)	299 (6.6%)		3948 (93.4%)	277 (6.6%)	
Female	2233 (94.0%)	142 (6.0%)		3641 (92.0%)	318 (8.0%)		3914 (92.6%)	312 (7.4%)	
Age group^a^			0.105			0.315			0.873
18–24 years old	1051 (95.1%)	54 (4.9%)		1147 (93.9%)	74 (6.1%)		1156 (93.1%)	86 (6.9%)	
25–34 years old	1724 (94.6%)	98 (5.4%%)		2076 (93.1%)	154 (6.9%)		1814 (93.6%)	124 (6.4%)	
35–44 years old	1386 (95.1%)	72 (4.9%)		2090 (92.5%)	169 (7.5%)		2195 (93.0%)	166 (7.0%)	
45–54 years old	766 (92.7%)	60 (7.3%)		1369 (92.4%)	113 (7.6%)		1409 (92.5%)	114 (7.5%)	
55–64 years old	338 (93.9%)	22 (6.1%)		784 (91.4%)	74 (8.6%)		832 (93.1%)	62 (6.9%)	
65 years and older	171 (91.9%)	15 (8.1%)		439 (93.0%)	33 (7.0%)		457 (92.5%)	37 (7.5%)	
Marital status^a^, ^b^			< 0.001			< 0.001			< 0.001
Never married	913 (16.8%)	58 (6.0%)		933 (93.1%)	69 (6.9%)		811 (93.6%)	56 (6.4%)	
Married	4350 (95.2%)	220 (4.8%)		6474 (93.9%)	424 (6.1%)		6535 (93.9%)	427 (6.1%)	
Widowed/divorced	172 (80.0%)	43 (20.0%)		497 (80.1%)	124 (19.9%)		517 (83.0%)	106 (17.0%)	
Educational attainment^a^, ^b^			0.778			0.876			0.506
No schooling	349 (94.6%)	20 (5.4%)		764 (91.6%)	70 (8.4%)		1338 (93.2%)	98 (6.8%)	
Incomplete primary	417 (94.6%)	24 (5.4%)		749 (92.4%)	62 (7.6%)		1232 (93.1%)	92 (6.9%)	
Completed primary	1451 (95.0%)	76 (5.0%)		2416 (92.9%)	184 (7.1%)		2854 (93.1%)	208 (6.8%)	
O level/Dakhil	722 (93.5%)	50 (6.5%)		1247 (93.3%)	90 (6.7%)		1069 (94.0%)	68 (6.0%)	
A level/Alim	948 (94.0%)	61 (6.0%)		1234 (93.0%)	93 (7.0%)		712 (91.2%)	64 (8.2%)	
Completed secondary/Fajil	711 (94.9%)	38 (5.1%)		808 (92.8%)	63 (7.2%)		401 (91.2%)	36 (8.2%)	
Post‐graduate	825 (94.1%)	52 (5.9%)		672 (92.7%)	53 (7.3%)		252 (92.0%)	22 (8.0%)	

^a^ Row percentages are in parentheses.

^b^ Three respondents refused to report their marital status/educational attainment and are omitted from the counts in these rows.

^c^ *p* values are based on chi‐square tests in which the null hypothesis assumes that the distribution of a given characteristic is similar in both groups.

## 3 Results

During the MPS, study interviewers called 506,659 mobile phone numbers, resulting in 22,731 complete interviews. Among respondents, 25.3% (*n* = 5757) were residents of large city corporations, 37.5% (*n* = 8522) were residents of other urban areas, and 37.2% (*n* = 8452) were rural residents. The proportion of respondents reporting at least one household death since 2020 was 5.6% in large city corporations (321/5757), 7.2% in other urban areas (617/8522), and 7.0% in rural areas (589/8452). The likelihood of reporting a recent death among household members was associated with the marital status of the respondent in all residential areas. For example, in large city corporations, 20.0% of respondents who were widowed or divorced reported a recent death in their household, compared to only 4.8% among respondents who were married at the time of the survey (*p* < 0.001). Other socio‐demographic characteristics of respondents did not differ between respondents with and without a death in their household during that timeframe.

MPS respondents reported 1527 deaths that had occurred since 2020 (Table 2), including 321 in large city corporations (21.0%), 617 in other urban areas (40.4%), and 589 in rural areas (38.6%). Across all residential strata, deaths were more common among men; for instance, in rural areas, 316 of 589 deaths (61.3%) occurred among males. The age distribution of deaths varied across residential strata (*p* = 0.009). Reported deaths were slightly younger in large city corporations than in rural areas. Most deaths occurred at home, though this proportion was notably higher in rural areas (72.5%) than in large city corporations (53.3%, *p* < 0.001). Burial places also varied, with community graveyards used more frequently in urban settings—45.3% in large city corporations and 47.1% in other urban areas—compared to 38.0% in rural areas (*p* = 0.012). In total, 99 of 1527 reported deaths (6.5%) had received a pre‐mortem diagnosis of COVID‐19, but this proportion was significantly higher (*p* < 0.001) in large city corporations (36/321, 11.2%) than in rural communities (19/589, 3.2%).

**TABLE 2:** Characteristics of deaths reported during MPS, by residence.

	Large city corporations, Dhaka and Chittagong (%)	Other urban areas (%)	Rural areas (%)	*p* ^b^	Total
*N*	321	617	589		1527
Gender of the deceased^a^				0.437	
Male	183 (57.0%)	373 (60.5%)	361 (61.3%)		917 (60.1%)
Female	138 (43.0%)	244 (39.5%)	228 (38.7%)		610 (39.9%)
Age at death				0.009	
0–4 years old	37 (11.5%)	43 (7.0%)	63 (10.7%)		143 (9.4%)
5–14 years old	4 (1.2%)	5 (0.8%)	6 (1.0%)		15 (1.0%)
15–24 years old	6 (1.9%)	12 (1.9%)	16 (2.7%)		34 (2.2%)
25–59 years old	70 (21.8%)	122 (19.8%)	102 (17.3%)		294 (19.3%)
60–79 years old	123 (38.3%)	289 (46.8%)	221 (37.5%)		633 (41.5%)
80 years and older	81 (25.2%)	146 (23.7%)	181 (30.7%)		408 (26.7%)
Marital status of the deceased				0.063	
Never married	49 (15.3%)	67 (10.9%)	83 (14.2%)		199 (13.1%)
Married	178 (55.5%)	370 (60.0%)	362 (61.8%)		910 (59.7%)
Widowed/divorced	94 (29.3%)	180 (29.2%)	141 (24.1%)		415 (27.2%)
Place of death				< 0.001	
Home	171 (53.3%)	373 (60.5%)	426 (72.5%)		970 (63.6%)
Health facility	132 (41.1%)	202 (32.7%)	116 (19.7%)		450 (29.5%)
Elsewhere	18 (5.6%)	42 (6.8%)	46 (7.8%)		106 (6.9%)
Place of burial				0.012	
Community graveyard	144 (45.3%)	289 (47.1%)	222 (38.0%)		655 (43.2%)
Family plot	161 (50.6%)	280 (45.6%)	313 (53.6%)		754 (49.7%)
Cremated	13 (4.1%)	41 (6.7%)	45 (7.7%)		99 (6.5%)
Other	—	4 (0.7%)	4 (0.7%)		8 (0.5%)
COVID‐19 diagnosis				< 0.001	
No	285 (88.8%)	573 (92.9%)	570 (96.8%)		1428 (93.5%)
Yes	36 (11.2%)	44 (7.1%)	19 (3.2%)		99 (6.5%)

^a^ Column percentages are in parentheses.

^b^ *p* values are based on chi‐square tests in which the null hypothesis assumes that the distribution of a given characteristic is similar in both groups.

In large city corporations and in other urban areas, approximately two thirds of deaths (Figure 1) reported during the MPS were also covered by at least one of the two potential ACMS (i.e., facility‐based or burial surveillance). In rural areas, this was the case for only one half of the reported deaths. In all residential areas, only a small number of deaths not covered by potential ACMS systems were covered by the case‐based surveillance of COVID‐19 deaths.

**FIGURE 1:**
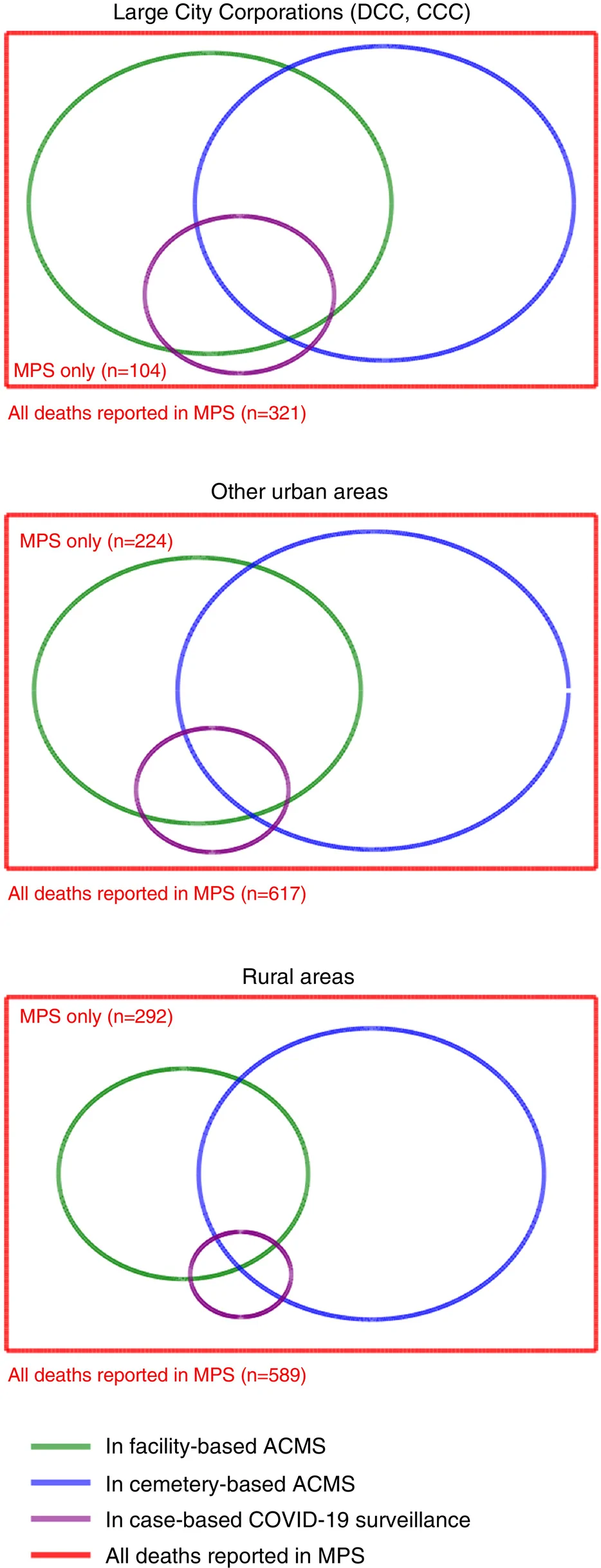
Venn diagram showing deaths reported during the MPS that are also covered by case‐based surveillance, and potential all‐cause mortality surveillance systems, by residential area.

In large city corporations, 104 out of 321 (32.4%, Table 3) reported deaths could only be detected by the MPS. In other urban areas, this was the case for 224 out of 617 reported deaths (36.3%). In rural areas, 292 out of 589 (49.6%) deaths reported during the MPS were uniquely listed by this data source and were not also covered by other potential ACMS systems, or by case‐based surveillance.

**TABLE 3:** Characteristics of deaths uniquely reported during the MPS, by residence.

	Large city corporations			Other urban areas			Rural areas		
	Potentially in ACMS and case‐based surveillance systems (%)	Only detected in MPS (%)	aOR (95% CI)^c^	Potentially in ACMS and case‐based surveillance systems (%)	Only detected in MPS (%)	aOR (95% CI)^c^	Potentially in ACMS and case‐based surveillance systems (%)	Only detected in MPS (%)	aOR (95% CI)^c^
*N* ^a^	217 (67.6%)	104 (32.4%)	—	393 (63.7%)	224 (36.3%)	—	297 (50.4%)	292 (49.6%)	—
Gender of the deceased^b^									
Male	116 (63.4%)	67 (36.6%)	Ref	250 (67.0%)	123 (33.0%)	Ref	190 (52.6%)	171 (47.4%)	Ref
Female	101 (73.2%)	37 (26.8%)	0.49 (0.27, 0.88)	143 (58.6%)	101 (41.4%)	1.24 (0.82, 1.88)	107 (46.9%)	121 (53.1%)	1.14 (0.78, 1.68)
Age at death^b^									
0–4 years old	31 (83.8%)	6 (16.2%)	0.20 (0.04, 1.09)	37 (86.0%)	6 (14.0%)	0.95 (0.19, 4.88)	42 (66.7%)	21 (33.3%)	0.66 (0.20, 2.10)
5–14 years old	3 (75.0%)	1 (25.0%)	0.33 (0.02, 5.11)	2 (40.0%)	3 (60.0%)	8.66 (0.87, 86.4)	4 (66.7%)	2 (33.3%)	0.67 (0.09, 5.08)
15–24 years old	4 (66.7%)	2 (33.3%)	0.73 (0.09, 6.15)	5 (41.7%)	7 (58.3%)	7.90 (1.43, 43.6)	10 (62.5%)	6 (37.5%)	0.81 (0.23, 2.82)
25–59 years old	52 (74.3%)	18 (25.7%)	Ref	92 (75.4%)	30 (24.6%)	Ref	58 (56.9%)	44 (43.1%)	Ref
60–79 years old	89 (78.4%)	34 (27.6%)	1.11 (0.54, 2.28)	101 (48.3%)	108 (51.7%)	1.74 (1.07, 2.83)	104 (47.1%)	117 (52.9%)	1.37 (0.84, 2.22)
80 years and older	38 (46.9%)	43 (53.1%)	2.89 (1.30, 6.45)	76 (52.1%)	70 (47.9%)	2.50 (1.44, 4.33)	79 (43.6%)	102 (56.4%)	1.46 (0.86, 2.48)
Marital status of the deceased^b^									
Never married	37 (17.1%)	12 (24.5%)	3.68 (0.79, 17.2)	51 (76.1%)	16 (23.9%)	0.51 (0.12, 2.13)	55 (66.3%)	28 (33.7%)	0.94 (0.32, 2.74)
Married	127 (58.5%)	51 (28.7%)	Ref	245 (66.2%)	125 (33.8%)	Ref	186 (51.4%)	176 (48.6%)	Ref
Widowed/divorced	53 (24.4%)	41 (43.6%)	1.82 (0.20, 0.69)	97 (53.9%)	83 (46.1%)	1.26 (0.80, 1.99)	56 (39.7%)	85 (60.3%)	1.34 (0.83, 2.17)

^a^ Row percentages are in parentheses.

^b^ Column percentages are in parentheses.

^c^ aOR = adjusted odds ratio; aORs were obtained from logistic regression models including all variables in this table as covariates; CI = confidence interval, ACMS = all‐cause mortality surveillance, MPS = mobile phone survey.

In large city corporations, deaths that could only be detected by MPS were less likely to be female deaths (aOR = 0.49, 95% CI = 0.26, 0.88), but they were more likely to have occurred among older age groups. For example, 53.1% of deaths at ages 80 years and older could only be detected by MPS versus 25.7% among deaths at ages 25 to 59 years old (aOR = 2.83, 95% CI = 1.30, 6.45). In other urban areas, while the likelihood that a death could only be detected by MPS was not associated with the gender of the deceased (aOR = 1.24, 95% CI = 0.82, 1.88), it was also associated with age at death. In particular, deaths in the 15–24 years old age group, and in the age groups above 60 years old were more likely to be listed only in the MPS than deaths at ages 25 to 59 years old. In rural areas, the likelihood that a death could only be detected by MPS and not covered by other potential mortality surveillance systems was not associated with observed socio‐demographic characteristics of the deceased.

## 4 Discussion

This paper examines the potential of MPS to complement other recommended methods of mortality surveillance in settings where the civil registration of deaths is limited, including (a) case‐based surveillance systems, which focus on deaths among individuals infected with a diagnosed infection, and (b) ACMS systems, which aim to count all deaths, irrespective of their cause. In Bangladesh, during the COVID‐19 pandemic, an MPS captured a substantial number of deaths that were likely missed by other existing and potential mortality surveillance systems. Many of the deaths listed during the MPS had occurred at home and had been buried on family‐owned plots of land. They were thus not covered by recommended ACMS systems that would focus on monitoring inpatients of health facilities or burials occurring in communal cemeteries. In this sample, the case‐based surveillance system set up during the COVID‐19 pandemic only marginally added to the coverage of potential facility‐based and cemetery‐based surveillance methods.

Among urban households in the MPS sample, approximately one third of the deaths listed during the MPS were not covered by potential mortality surveillance systems that could be established in health facilities or in communal cemeteries. Deaths not covered by these potential ACMS systems included in particular an increased number of deaths that had occurred among males, as well as among young adults and the elderly. In rural areas, close to half of the deaths listed during the MPS were not covered by other potential mortality surveillance systems. Our work thus indicates that MPS might help fill large gaps in recommended approaches to monitor mortality, particularly in under‐served populations, and among social groups less connected to the health system (i.e., males, youth).

Our findings have several limitations. First, our analyses compared MPS data to those that may be generated by hypothetical ACMS systems. We thus considered that all the deaths reported to have occurred in health facilities, or to have been buried in communal cemeteries, would be recorded by such ACMS systems. This is a strong assumption. When implemented, ACMS systems may not be complete, for example if record‐keeping procedures are imperfect, if they are not sufficiently staffed t0 continuously record events, and/or if they only selectively cover a subset of health facilities or burial grounds. In real‐world settings, the proportion of deaths uniquely covered by MPS might be higher than estimated in this study.

Second, contrary to our assessment of ACMS systems, our analyses do not compare MPS data with those generated by a hypothetical case‐based surveillance system; rather, they use data reported by respondents on pre‐mortem diagnoses obtained during the COVID‐19 pandemic. This comparison with proxy‐reported data might be affected by respondent errors. Some household informants might have experienced stigma related to COVID‐19 and preferred not to disclose the potential COVID‐19 diagnosis of their deceased relative(s) during the phone interview. In such cases, our analyses underestimate the coverage of case‐based surveillance and inflate the potential benefits of MPS. In other cases, a pre‐mortem diagnosis of COVID‐19 might not have been included in the counts of deaths generated by case‐based surveillance. For example, the introduction of rapid self‐testing kits for SARS‐CoV‐2 might have led to a number of positive tests that were not later reported to health authorities.

Third, our data do not allow investigating which deaths are missed by an MPS, and among those, how many might be covered by other potential systems such as those focused on health facilities or cemeteries. In particular, MPS may miss deaths among households that do not have access to a mobile phone [61, 62, 63]. According to the most recent household surveys in Bangladesh, there are only a few households left without access to a mobile phone [54], but they might be concentrated in areas with high levels of poverty and mortality. Similarly, MPS that rely on random digit dialling often have low response rates. In this study in Bangladesh, approximately 5% of the mobile numbers called by interviewers yielded a complete interview, similar to or only slightly lower than participation rates observed in MPS that used random digit dialling procedures in other LMICs [59, 64, 65]. Our data might thus have missed a number of deaths that had occurred in households where mobile phones are available, but where potential respondents could not be reached or refused to participate. Various methods, however, exist that might help increase the representativeness of MPS samples [66].

Fourth, the data generated by MPS might be less reliable than data collected from other modalities [67]. MPS respondents might inaccurately report data on recent deaths in their households [68]. Some might not want to disclose such events to unknown callers. Others might misstate the age at which a death occurred, the date of a death, or they might mistakenly include family members who did not reside in their household at the time of death. Conversely, respondents might not include deaths that have occurred to more vulnerable members of the household, such as domestic workers. Deaths that occur in single‐person households are also necessarily excluded from the list of deaths reported by survivors during MPS. In some cases, deaths that occur in larger households might also be excluded if the death of a member precipitated the dissolution of the household. By comparison, other potential mortality surveillance systems, such as those implemented in health facilities or at cemeteries, might cover such deaths.

For these reasons, additional validation studies of the mortality data generated by MPS in LMIC settings are needed. Similar to validation studies of the mortality data collected during in‐person survey [69, 70], such studies would require detailed record linkages between multiple data sources in order to characterize (a) which deaths are omitted from MPS, and (b) how accurate are MPS respondents in reporting the age at death, time since the death, and other circumstances of the deaths of their relatives. In Bangladesh, multiple opportunities exist to conduct record linkages between MPS and other mortality records, since several surveillance systems are in place throughout the country. These include, for example, HDSS data collected in several administrative areas, burial surveillance data from Dhaka and Chittagong, and nationwide SRS data.

Despite limitations and the need for additional validation, our work highlights the potential utility of MPS in mortality surveillance in Bangladesh and other LMICs. Not only are the logistics required to set up and implement MPS greatly simplified, in principle, MPS may also cover key gaps in the data generated by other data collection and surveillance methods, including in times of crises such as epidemics or conflicts.

## Funding

This study was made possible with financial support from the Bill and Melinda Gates Foundation (INV‐023211). The funder had no role in study design, data collection, data analysis, data interpretation, or writing of the report.

## Ethics Statement

The study protocol was reviewed by the institutional review boards at Johns Hopkins University School of Public Health (IRB00017284) and by the Institutional Review Board of the Institute of Epidemiology, Disease Control and Research. All participants provided oral consent for the survey.

## Conflicts of Interest

The authors declare no conflicts of interest.

## Data Availability

The data that support the findings of this study are available on request from the corresponding author. The data are not publicly available due to privacy or ethical restrictions.
